# Whole genome resequencing and complementation tests reveal candidate loci contributing to bacterial wilt (*Ralstonia* sp.) resistance in tomato

**DOI:** 10.1038/s41598-022-12326-x

**Published:** 2022-05-19

**Authors:** Derek W. Barchenger, Yu-ming Hsu, Jheng-yang Ou, Ya-ping Lin, Yao-cheng Lin, Mark Angelo O. Balendres, Yun-che Hsu, Roland Schafleitner, Peter Hanson

**Affiliations:** 1 World Vegetable Center, Tainan, Taiwan; 2 CNRS, INRAE, Institute of Plant Sciences Paris-Saclay (IPS2), Univ Evry, Université Paris-Saclay, 91405 Orsay, France; 3 Biotechnology Center in Southern Taiwan, Agricultural Biotechnology Research Center, Academia Sinica, Tainan, Taiwan; 4 Institute of Plant Breeding, College of Agriculture and Food Science, University of the Philippines Los Baños, Los Baños, Laguna Philippines

**Keywords:** Plant breeding, Genetics

## Abstract

Tomato (*Solanum lycopersicum*) is one of the most economically important vegetable crops worldwide. Bacterial wilt (BW), caused by the *Ralstonia solanacearum* species complex, has been reported as the second most important plant pathogenic bacteria worldwide, and likely the most destructive. Extensive research has identified two major loci, *Bwr-6* and *Bwr-12*, that contribute to resistance to BW in tomato; however, these loci do not completely explain resistance. Segregation of resistance in two populations that were homozygous dominant or heterozygous for all *Bwr-6* and *Bwr-12* associated molecular markers suggested the action of one or two resistance loci in addition to these two major QTLs. We utilized whole genome sequence data analysis and pairwise comparison of six BW resistant and nine BW susceptible tomato lines to identify candidate genes that, in addition to *Bwr-6* and *Bwr-12,* contributed to resistance. Through this approach we found 27,046 SNPs and 5975 indels specific to the six resistant lines, affecting 385 genes. One sequence variant on chromosome 3 captured by marker Bwr3.2dCAPS located in the *Asc* (*Solyc03g114600.4.1*) gene had significant association with resistance, but it did not completely explain the resistance phenotype. The SNP associated with Bwr3.2dCAPS was located within the resistance gene *Asc* which was inside the previously identified *Bwr-3* locus. This study provides a foundation for further investigations into new loci distributed throughout the tomato genome that could contribute to BW resistance and into the role of resistance genes that may act against multiple pathogens.

## Introduction

Tomato (*Solanum lycopersicum* L.) is widely grown and one of the most economically important vegetable crops worldwide. Global production of tomatoes has continuously increased for the past 50 years, especially in tropical and subtropical regions. Tomato crops can be infected by disease-causing bacterial, fungal, and viral pathogens that can reduce yield, fruit quality, shelf-life, and nutritional content. Bacterial wilt (BW), caused by the *Ralstonia solanacearum* species complex (RSSC), is one of the most destructive plant pathogenic bacteria^1^. The RSSC is favored by high temperatures and humidity, and, as extreme weather events become more frequent and severe through climate change, it is anticipated that BW will become more common and destructive. Management of BW with pesticides is not a viable option because the pathogen survives in the soil for many years and has a wide host range^2^. Other management strategies include soil solarization, which is of limited effectiveness due to the existence of the pathogen deep in the soil. An integrated approach has been identified as the best way to manage the disease, including irrigation management, grafting, crop rotation, sanitation (removing weeds and plant debris and also cleaning farm equipment), and managing insect and nematode pests. Host resistance is the single most effective management strategy associated with BW^3^ and planting resistant cultivars is the cheapest, simplest, and most environmentally friendly approach to limit losses^4^. Sources of resistance to BW originating from cultivated tomato and its close wild relatives, *S. pimpinellifolium* and *S. lycopersicum* var. *cerasiforme,* have been identified, but none are immune and expression of resistance is strongly influenced by pathogen strain, temperature, soil pH and the interactions among these factors^3^. Furthermore, BW resistance has been associated (linked) with small fruit weight, bitter flavor, susceptibility to root-knot nematodes, and other negative traits^5^. Variable reaction of BW resistance sources^6^ coupled with quantitative inheritance of resistance complicates conventional breeding and development of resistant cultivars.

A coordinated multilocation testing of a set of resistance sources by a team of collaborators following comparable testing and evaluation protocols identified ‘Hawaii 7996’ (H7996) as one of the most stable resistance sources with a high survival rate across 12 field trials in 11 countries^7^. Later, INRA-CNRS, University of the Philippines Los Baños, and the World Vegetable Center (WorldVeg) developed an advanced recombinant inbred line (RIL) population (188 F_9_ lines) derived from the cross of H7996 by susceptible *S*. *pimpinellifolium* ‘West Virginia 700’ (WVa700). Multi-location testing of this mapping population in nine trials, seven in Asia and two in Reunion Island, revealed the presence of two major genomic regions (*Bwr-6* and *Bwr-12*) conditioning BW resistance, as well as additional QTLs with minor or strain-specific effects^8^, supporting the findings of Carmeille et al.^9^ who reported major QTLs on chromosome 6 (*Bwr-6*) and minor QTLs on chromosomes 3, 4, and 8 (*Bwr-3*, *Bwr-4*, and *Bwr-8*, respectively). The molecular markers developed for the selection of *Bwr-6* and *Bwr-12* QTLs are certainly useful^4,9–11^; however, they do not completely explain the resistant phenotype and have some level of mismatch resulting in false positives and selection of susceptible individuals^12^.

The QTL *Bwr-12*, located in a 2.3-cM interval of chromosome 12, accounted for much of the phenotypic variation for resistance to phylotype I isolates (recently reclassified as *R. pseudosolanacearum*)^12^. Virus-induced gene silencing assays suggested the involvement of leucine-rich repeat receptor-like kinases *Solyc12g009520* and *Solyc12g009550* located in the *Bwr-12* QTL interval with resistance to phylotype I strains^13^. Through whole genome resequencing, Kim et al.^14^ identified four genes that encode putative leucine-rich repeat receptor-like proteins that were associated with resistance to BW on chromosome 12. The authors reported one SNP marker in the gene *Solyc12g009690.1* that could be tightly linked to *Bwr-12*. However, in our analysis this marker does not improve selection accuracy for BW resistance beyond previously developed molecular markers linked to the trait (unpublished data). The QTL *Bwr-6* encompasses a 15.5-cM region on chromosome 6 that may include one or more important QTLs for resistance to phylotype II isolates (classified as *R. solanacearum*) as well as more broad-spectrum resistance^12^. *Bwr-6* is a large region and molecular markers in these regions do not completely explain the broad-spectrum resistance in the offspring of ‘H7996^14^. Recent efforts focused on fine-mapping the *Bwr-6* and *Bwr-12* regions to identify important resistance loci and closely linked markers have been promising^15^. The authors identified four QTLs associated with strain-specific resistance on chromosome 6 and three on chromosome 12, explaining 14–54% of the overall variability. For validation, they used a set of 80 near-isogenic lines (NILs) derived from the RILs developed by Wang et al.^8^ and found significant association with the phenotype^15^. Field trials of H7996 and WorldVeg tomato lines homozygous for *Bwr-12* and *Bwr-6* under BW pressure in Benin revealed that the WorldVeg lines did not demonstrate high levels of resistance like H7996^16^. This result suggests that H7996 carries additional major BW QTL besides *Bwr-12* and *Bwr-6*. The objective of this study was to identify loci contributing to BW resistance besides *Bwr-6* and *Bwr-12* to support breeding for more durable resistance in tomato varieties.

## Results and discussion

### Disease resistance levels among tomato lines

None of the lines had complete resistance to both pathogen strains (Pss4 and Pss1632) in these trials, including H7996, the best-known tomato resistance source (Table 1). Wilting can occur in BW resistant tomato lines, the extent of which depends on pathogen strain, temperature, and other environmental conditions^12,17,18^. However, the proportion of wilted plants in resistant lines was usually less than in susceptible lines (Table 1). The six lines in the resistant group selected for whole genome sequencing had higher levels of resistance to both pathogen strains (average of 95 and 83% resistance to Pss4 and Pss1632, respectively) compared to the performance of the nine susceptible lines (average of 28 and 19% resistant plants for Pss4 and Pss1632, respectively) (Table 1). Both groups typically had slightly higher levels of resistance to Pss4 than Pss1632. Within the susceptible group, there were large differences in symptom expression between and within pathogen strains. TBL-2, Pant Bahar, and L390 were highly susceptible to both strains. CRA84-23–1 115 was highly resistant to Pss4 (90% resistant) but highly susceptible to Pss1632 (10% resistant) (Table 1). CRA84-57-1 140, T-245, and ST/2 had moderately low levels of resistance to both strains (Table 1). These results support the extensive body of literature highlighting the complexity of host-pathogen interactions in the tomato-BW pathosystem, as reviewed by Hayward et al.^3^. Furthermore, the higher level of virulence of Pss1632 was previously reported^12^. When challenged with Pss4, LS-89 and F7 80 Pink were the most resistant accessions (100% resistant), while Pant Bahar, L390, and LA3501 were the most susceptible (0%) (Table 1). The accession F_7_ 80-465-10-pink was the most resistant to Pss1632 (92.5%), while TBL-2 was the most susceptible (100% of symptomatic plants) (Table 1). The resistant and susceptible reactions of the accessions screened in this study were generally in alignment with the previous work of Kunwar et al.^12^ employing a partly overlapping set of materials. Hai et al.^17^reported that LA3501 was resistant to BW strain Pss186 but susceptible to Pss4. Strain- and environment-specific reactions have been previously reported^8, 12^ and these will likely limit the development of widely applicable molecular markers associated with BW resistance. To account for the variability of resistance in the accessions, only the five most resistant or most susceptible individual plants per accession were selected for sequencing and downstream analysis.

**Table 1:** Average resistance percentage of the highly resistant and highly susceptible tomato lines used for sequencing two weeks after inoculation with two different strains of *Ralstonia* sp, Pss4 (race 1, biovar 3, *R*. *pseudosolanacearum*) and Pss1632 (race 3, biovar 2, *R*. *solanacearum*), during the hot season (June–July) in 2018. Five individual plants with extremes in the phenotype (highly susceptible early in the evaluation, highly resistant late in the evaluation) were selected for sequencing.

Tomato line	Country of origin	Resistant percent screened against Pss4	Resistant percent screened against Pss1632	Average percent resistance
LS-89	Japan	100	85	92.5
Hawaii 7997	USA	95	82.5	88.8
F_7_ 80-465-10-pink	Philippines	85	92.5	88.8
F_7_ 80 pink	Philippines	100	72.5	86.3
Hawaii 7996	USA	95	75	85
LE415 Anagha	India	95	90	82.5
CRA84-23-1 115	Guadeloupe	90	15	52.5
CRA84-57-1 140	Guadeloupe	60	30	45
T-245	Sri Lanka	40	35	37.5
S/T2	Philippines	30	35	32.5
Rodade	South Africa	20	25	22.5
LA3501	USA	0	20	10
TBL-2	France	10	0	5
L390	Taiwan	0	10	5
Pant Bahar	India	0	5	2.5

### Whole genome sequencing of 15 tomato varieties for genome wide variant detection

The read depth of the sequencing ranged from 24.7 × (LE415 Anagha) to 56.8 × (H7997), with an average read depth of 38.6 × (Table 2). Genome coverage and properly mapped pair-end reads were always greater than 98% in our experiment (Table 2). When compared to the ‘Heinz 1706’ annotated genome (v. SL4.0), we identified an average of 883,682 SNPs and 222,565 indels. LS-89 had the greatest number of SNPs, at 1,643,618 followed by LA3501 with 1,637,262, while the greatest number of indels were identified for LA3501 (Table 2). The highly susceptible cultivar Pant Bahar had the fewest number of SNPs and indels with 359,227 and 157,239, respectively (Table 2). The number of polymorphisms identified in our study is in line with several other studies using different accessions of domesticated tomato species^19–21^, which was generally fewer than 2 million SNPs, although results were based on different versions of the ‘Heinz 1706’ reference genome.

**Table 2:** Summary statistics of the sequence quality, coverage and polymorphisms of the bacterial wilt (Pss4 (race 1, biovar 3, *Ralstonia pseudosolanacearum*) and Pss1632 (race 3, biovar 2, *R*. *solanacearum*) resistant and susceptible tomato lines.

Tomato line	Estimated read depth	Genome coverage ratio (%)	Properly mapped paired reads (%)	All SNPs	All InDels	Homozygous SNPs	Homozygous InDels	Phenotypic response
F7 80-465-10-pink	46.4	99.2	99.2	529,584	207,522	327,246	166,770	R
LE415 Anagha	24.7	98.9	99.5	410,103	172,062	157,920	135,478	R
LS 89	35.3	98.7	98.7	1,643,618	303,559	1,327,260	251,884	R
Hawaii 7997	56.8	98.8	99.0	876,848	223,157	634,321	181,368	R
Hawaii 7996	34.2	98.9	98.9	1,136,702	247,511	849,093	201,316	R
F7 80 pink	44.1	99.4	99.3	534,965	213,438	327,984	168,569	R
TBL-2	41.7	99.2	99.3	627,186	196,732	352,923	155,867	S
Pant Bahar	25.3	98.6	99.4	359,227	157,239	136,709	126,529	S
L390	32.5	99.5	99.0	397,321	185,729	225,619	154,056	S
CRA84-23-1 115	26.4	98.6	99.0	991,748	221,898	602,564	170,932	S
LA3501	27.8	98.4	98.6	1,637,262	315,105	1,331,932	263,758	S
Rodade	26.5	99.1	99.2	606,730	192,083	392,549	159,238	S
CRA84-57-1 140	53.0	99.3	99.3	689,382	220,246	219,025	153,402	S
T-245	51.9	99.6	99.2	1,023,995	244,588	113,967	130,427	S
S/T2	52.7	99.0	99.0	1,040,560	237,605	775,087	191,734	S

**Figure 1:**
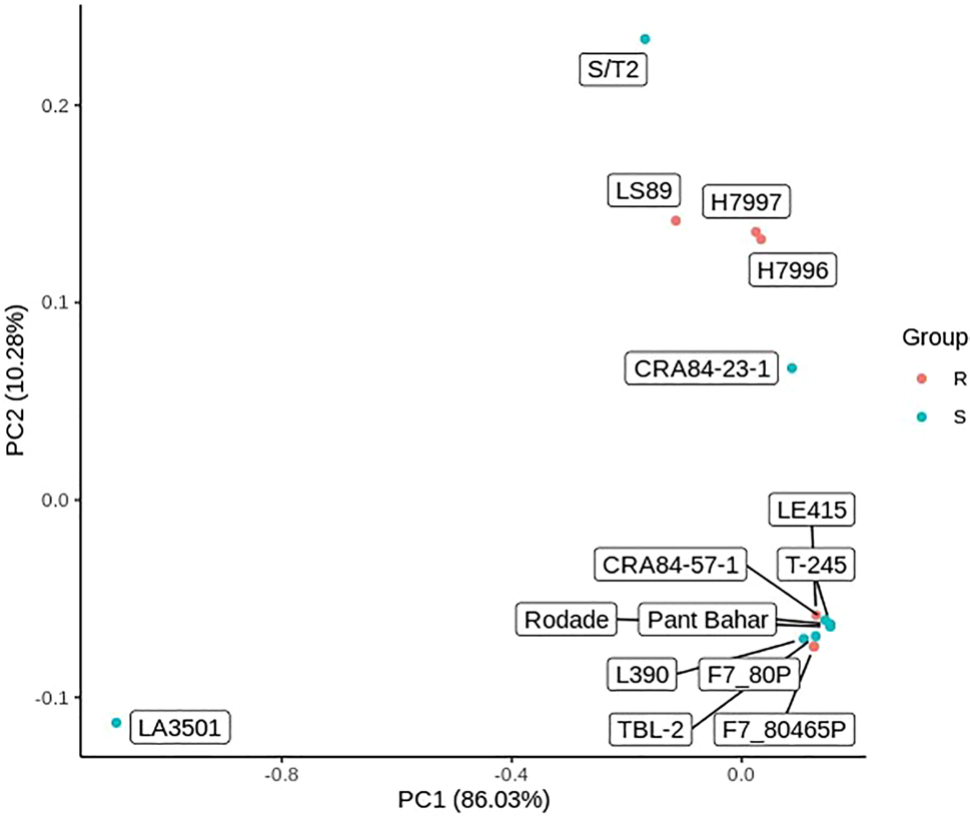
The Principal Coordinate Analysis based on all of the high-quality polymorphisms of the bacterial wilt (*Ralstonia* sp.) resistant (R; red) and susceptible (S; blue) tomato lines. H7796 is Hawaii 7996 and H7997 is Hawaii 7997.

Three resistant and six susceptible accessions (F7_80P, F7_80465P, CRA84-57-1, L390, LE415, Pant Bahar, Rodade, T-245, and TBL-2) formed a distinct cluster based on similarities in the high-quality SNPs identity in this study (Fig. 1). However, the highly unique and BW susceptible line LA3501 had a strong interactive force on the other accessions, which could make this cluster of lines appear more similar than they actually were. LA3501 contains an introgression on chromosome 6 derived from *S*. *pennellii* which provides strain-specific BW resistance^17^; this DNA fragment probably contributed to the genetic uniqueness of this line compared to most other lines in our study. We found that H7996 and H7997 were genetically similar while the other accessions in our study appeared more unique (Fig. 1).

**Figure 2:**
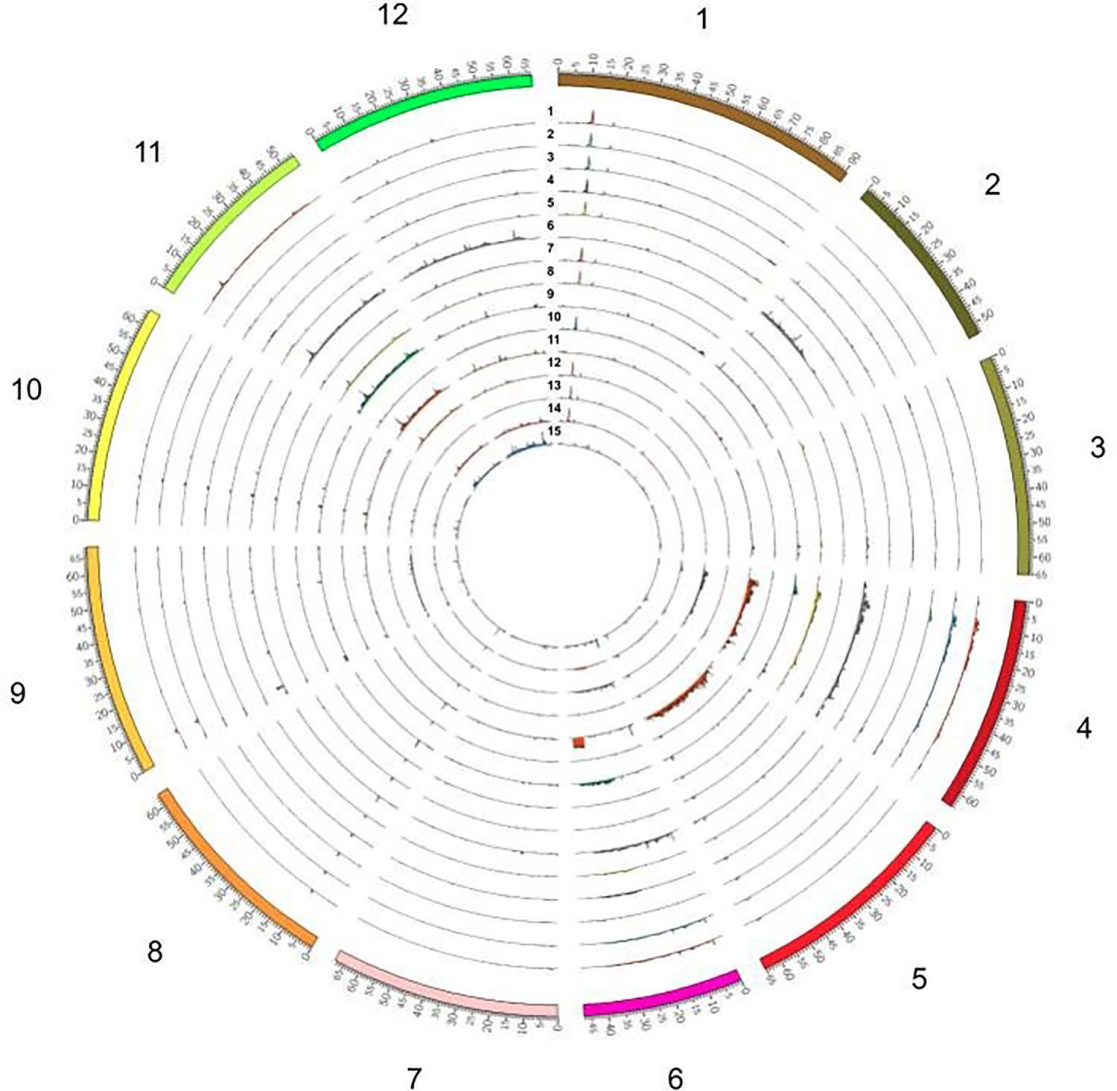
The distribution of SNPs across the genome for 15 bacterial wilt (*Ralstonia* sp.) resistant and susceptible tomato lines. The histograms represent the number of SNPs in 100-kb for the 15 tomato accessions. The lines are numbered (1) Hawaii 7996, (2) Hawaii 7997, (3) LE415, 4) F7_80P, (5) F7_80465P, (6) LS89, (7) Bahar, (8) CRA84_115, (9) CRA84_140, (10) L390, (11) LA3501, (12) Rodade, (13) ST2, (14) T_245, and (15) TBL_2.

We compared the SNP distribution of all accessions, and found that the six resistant accessions had higher SNP density in the regions around *Bwr-6* and *Bwr-12* than the nine susceptible accessions (Fig. 2 and Supplemental Fig. 1). However, we also observed that resistant and susceptible lines shared many regions with similar SNP distribution (Fig. 2). Since our objective was to identify loci that contribute to BW resistance not explained by *Bwr-6* and *Bwr-12*, those regions with similar SNP distributions common in resistant and susceptible accessions were removed from further consideration as candidates for discovery of new resistant loci. To comprehensively screen the candidate polymorphisms that contributed to resistance, we compared each resistant accession with all nine susceptible accessions, and removed SNPs that were identified in any of the susceptible accessions. This comparison allowed us to extract variants that are uniquely found in each resistant line but not in any of the susceptible lines.

**Figure 3:**
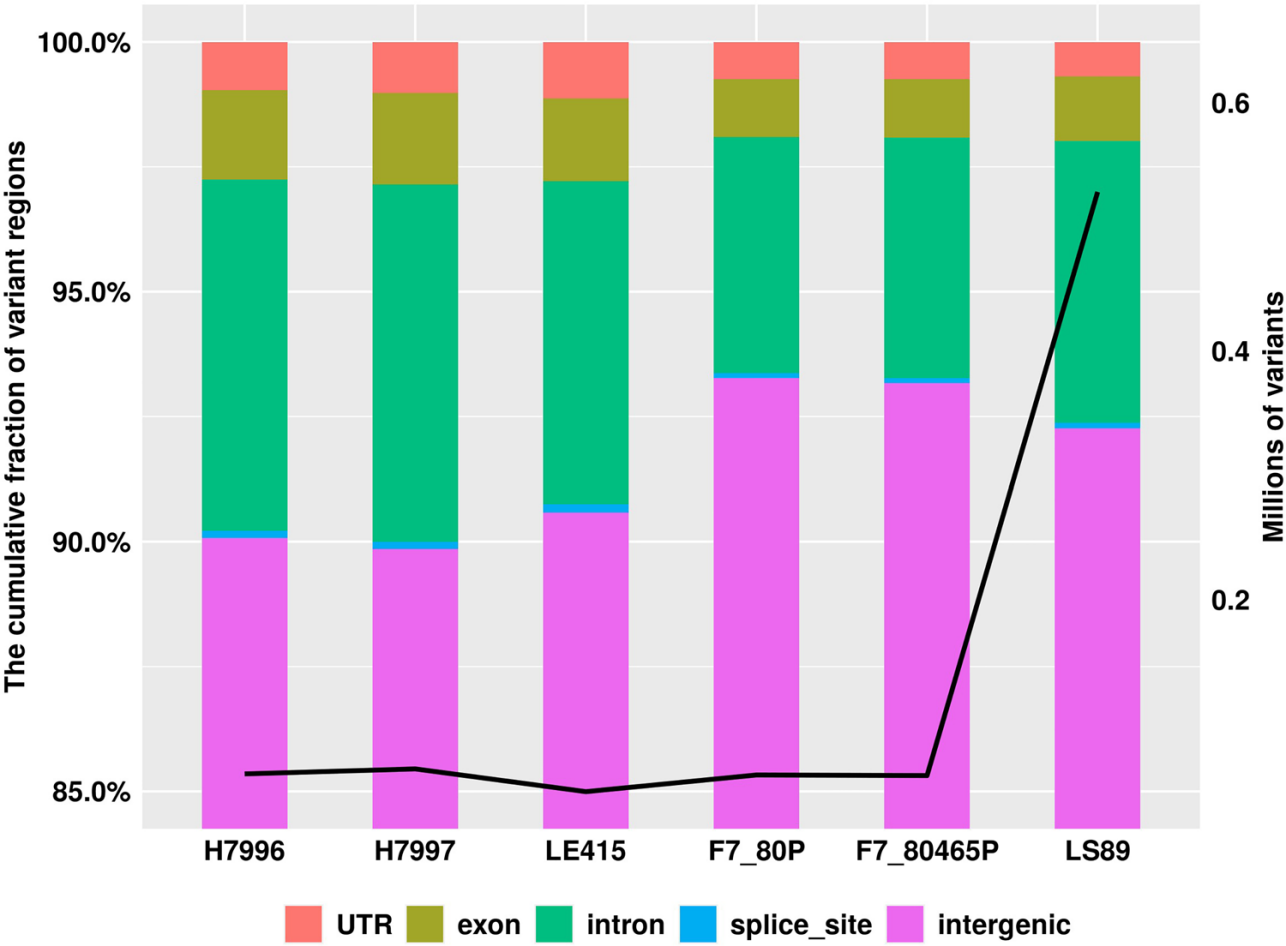
The proportion and number of SNPs acquired by genomic features of the six highly bacterial wilt (*Ralstonia* sp.) resistant tomato lines. The bars represent the proportion of genomic features in which SNPs of tomato lines are located, and the black line is the number of SNPs contained in each of the tomato lines. In the legend, “UTR” includes 5′UTRs and 3′UTRs, and “splice_site” includes the donors, receptors and regions of splice sites.

**Figure 4:**
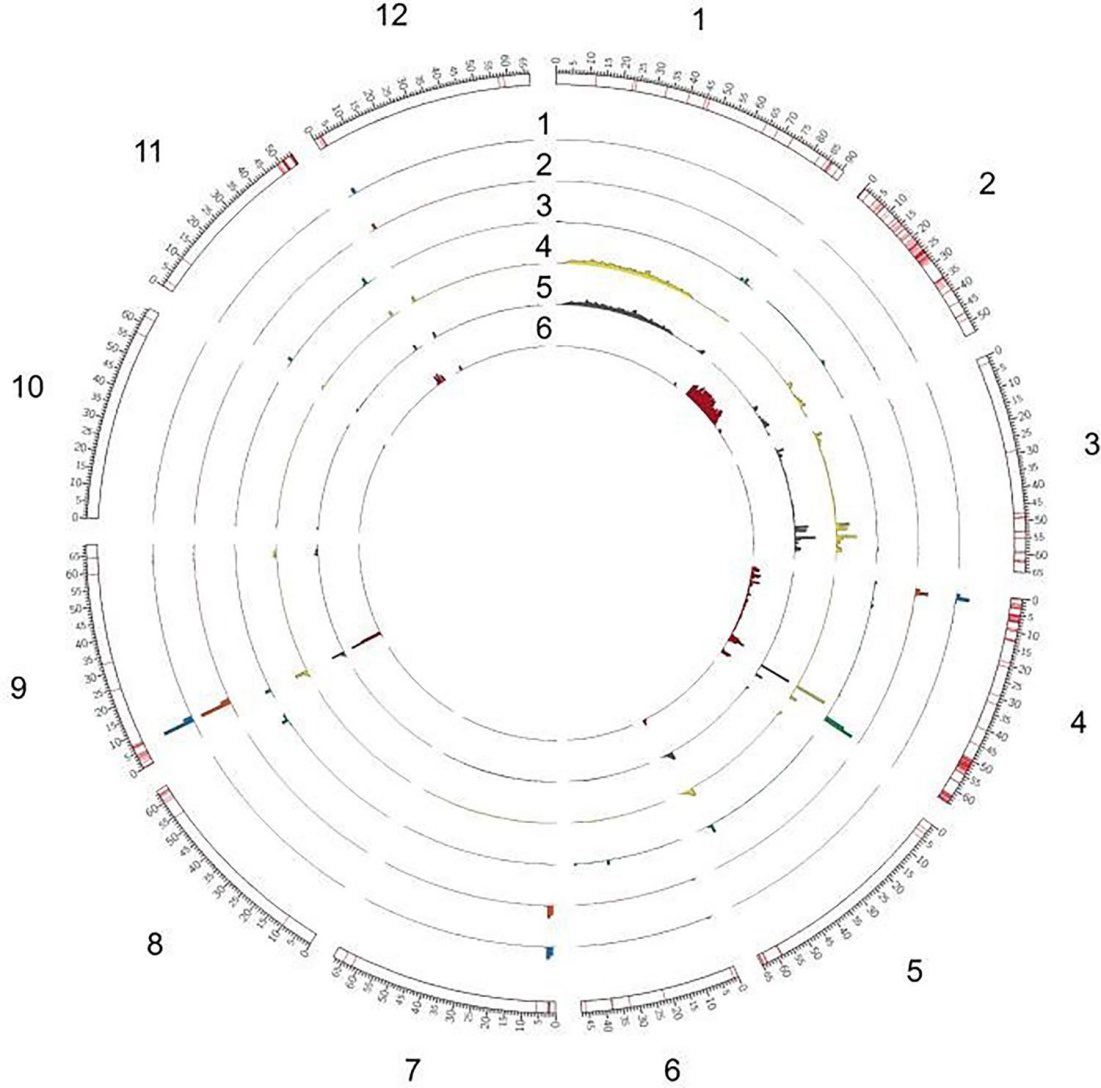
The genome-wide distribution of filtered variants and highly-affected genes of six bacterial wilt (*Ralstonia* sp.) resistant tomato lines. The 12 chromosomes are numbered clockwise, and the red bands on the outermost bars are genes highly affected by polymorphisms of 6 resistant accessions. The six histograms display the number of SNPs in 1-mb windows of 6 resistant tomato accessions. The lines are numbers (1) Hawaii 7996, (2) Hawaii 7997, (3) LE415, (4) F7_80P, (5) F7_80465P, and (6) LS89.

In the first stage of comparison, we retained only homozygous polymorphisms for further analysis. The accessions had an average of homozygous 518,279 SNPs and 174,088 indels (Table 2). Then, we compared each of the six resistant lines individually with all nine of the susceptible lines and retained variants that were uniquely identified in resistant lines. With these two filters, only about 8% of total variants of resistant accessions were retained. Among the resistant accessions, LS-89 had the greatest number of unique variants with 313,359 SNPs and 42,444 indels, while the other resistant accessions have an average of 27,046 unique SNPs and 5,975 unique indels (Fig. 3). Kim et al.^14^ conducted a similar analysis using two susceptible and seven resistant accessions, including H7996, for comparison and found 5,259 SNPs to be polymorphic between resistant and susceptible groups. LS-89 is a BW-resistant rootstock cultivar developed in Japan originating as a selection from either H7996^22^ or H7998^23^, although both H7996 and H7998 were reported to originate from the same source (PI 127805A)^24^. However, it is possible that H7996, H7997 and several other Hawaii-prefixed lines were selections out of a genetically diverse accession ‘HSBW’ (Hot Set Bacterial Wilt)^25^. LS-89 should not differ greatly from H7996 but we found that LS-89 was genetically distinct from H7996, H7997 and the other resistance sources in our experiment (Fig. 4) although it was not compared with H7998 which was not included in our analysis. LS-89 might be derived from a different HSBW selection but since this original source is lost, no follow-up is possible. There is a chance that the seed source held by the World Vegetable Center is incorrect, despite it having a similar resistance reaction as the original LS-89^26^.

### Comparison of WGS variants with QTL mapping

**Figure 5:**
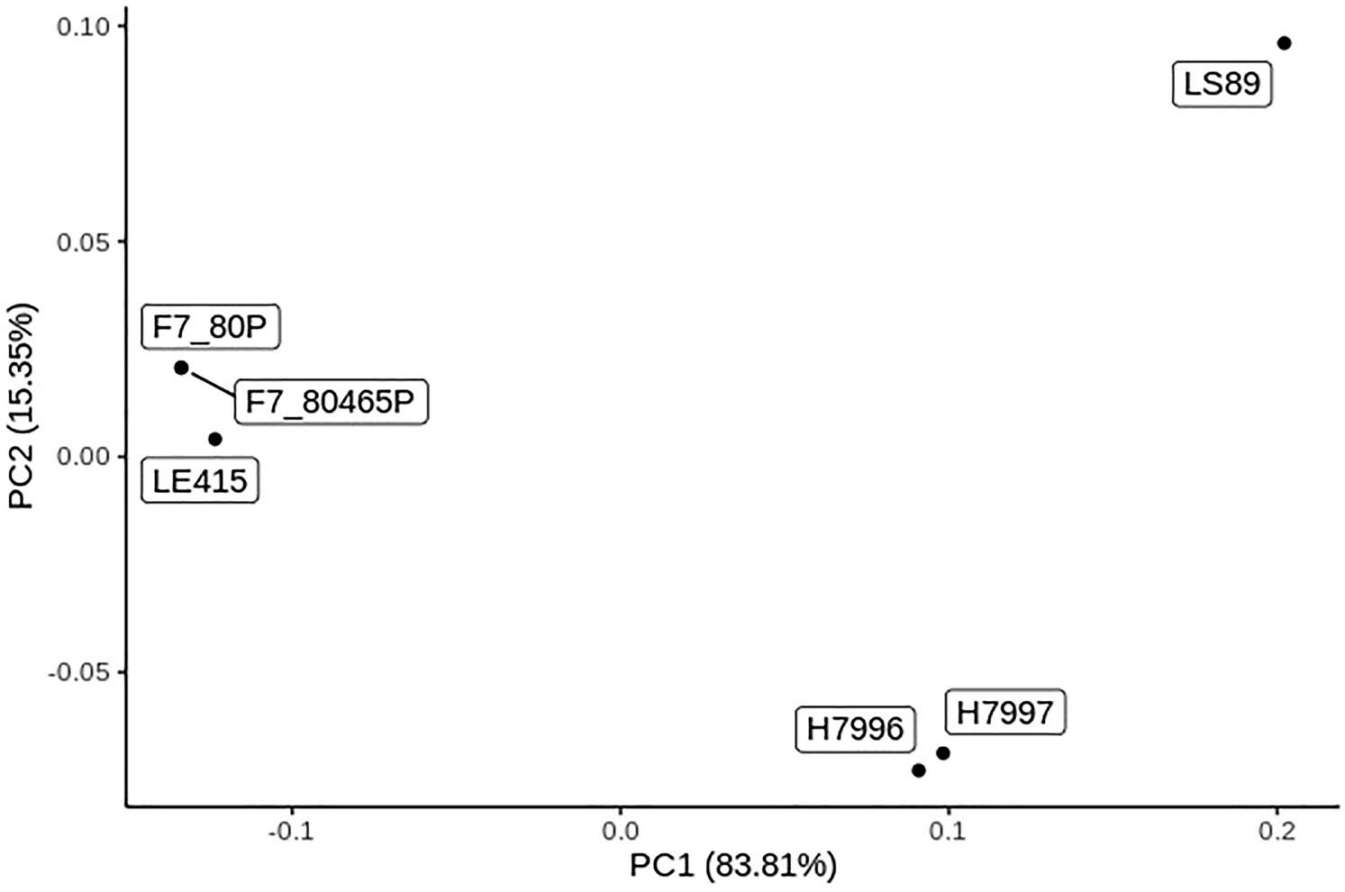
The Principal Coordinate Analysis based on the polymorphisms of the six bacterial wilt (*Ralstonia* sp.) resistant tomato lines used in this study. F7_80P and F7_80465P share the same PC1 and PC2. H7996 is Hawaii 7996 and H7997 is Hawaii 7997.

Based on these polymorphisms specific to resistant lines, we compared them among the 6 resistant lines and previous studies that identified QTLs associated with the bacterial wilt resistance. The proportion of common polymorphisms among the resistant tomato lines varied across the chromosomes (Fig. 4). Only two polymorphisms on chromosome 12 were common among all six resistant lines (Fig. 4), which were near but not within the previously identified resistance QTL *Bwr-12*^8,14^. The number of unique polymorphisms were high and ranged from 196,901 on chromosome 2 to 1,429 polymorphisms on chromosome 10 (Fig. 4). There were 25 polymorphisms that were common among 5 of the 6 resistant lines and 66 polymorphisms that were common among 4 of the resistant lines (Fig. 5), all of which were within the region previously identified by Kim et al.^14^ and near the large resistance QTL *Bwr-6* (22.2–39.6 Mb)^8^. Multiple QTLs within the large *Bwr-6* and *Bwr-12* loci have been previously reported^15^; therefore, the common polymorphisms on chromosomes 6 and 12 found here warrant further investigation as they could be within candidate genes contributing to resistance that are linked to the major QTLs *Bwr-6* and *Bwr-12* but have not yet been fully characterized. The majority of the unique polymorphisms were from LS-89 (Fig. 3), which underlies the genetic distinctiveness of this line (Fig. 5). Interestingly, we found that our other resistance sources form two distinct clusters based on genetic similarity, with H7996 and H7997 being similar and with F7_80P and F7_80465P being extremely similar and clustering closely with LE415 (Fig. 4). This genetic structure could be a contributing factor in the overall lack of common polymorphisms in our study and a preponderance of polymorphisms that were common among only two or three sources.

We then predicted the functional effects of variants uniquely identified in 6 resistant lines targeting protein-coding genes. The vast majority of the variants were detected in intergenic or intronic regions (Fig. 3), with fewer than 1,000 SNPs being located in genic regions in most entries with the exception of LS-89, which contained 6,500 SNPs in protein-coding regions (Supplemental Table 1). For the variants in UTR, the 3′UTRs had 1.64 to 2.65 times more variants than 5′UTRs. The ratio of nonsynonymous and synonymous mutation ranged from 0.56 to 0.94. Frameshift mutations were the most frequent type of mutation we identified (Supplemental Table 1).

The details of candidate genes are provided in Supplemental Table 2. A large number of polymorphisms were unique to LS-89 and not present in the other resistant lines. In total, we found high impact mutations specific to the six resistant lines in 385 genes. The polymorphisms identified here were not uniformly distributed among the 12 chromosomes and most were located on chromosomes 2 and 4 (Fig. 4 and Supplemental Fig. 2). Using H7996, Kim et al.^14^ found 265 resistant-specific SNPs located in coding regions, with most SNPs located on chromosomes 6 and 12 near *Bwr-6* and *Bwr-12* QTLs.

As expected, the three parental lines (CLN3641F1-5-11-14-4-25-20-11-7(F), CLN4018F1-6-7U14-29-21-14-5 and H7996) were resistant against BW strain Pss4 used in our experiment. Based on molecular marker results, all F_2_ plants in both mapping populations had either the homozygous dominant or heterozygous alleles at *Bwr-6* and *Bwr-12*, as did the three parental lines (Supplemental Table 3). The two F_2_ populations showed different segregation patterns for inheritance of resistance to Pss4 strain: CLN4397-4 did not deviate significantly from a 3:1 (resistant to susceptible) ratio while CLN4398-8 showed a 9:7 ratio (Table 3). Given that the populations were homozygous for both *Bwr-6* and *Bwr-12*, there were apparently two additional independent loci contributing to resistance in CLN4398-8 and one additional independent locus in CLN4397-4. The role of multiple loci or complex inheritance patterns associated with resistance to BW in tomato has been widely reported^8,15,27–33^, which supports our findings. However, one study identified a single dominant gene conferring resistance to BW in H7996^34^ and H7998^35^. The difference in findings is not necessarily contradictory but could be due to different pathogen strains used for screening in inheritance studies.

**Table 3:** Goodness of fit test for inheritance of resistance to the Pss4 isolate of bacterial wilt (race 1, biovar 3, *Ralstonia pseudosolanacearum*) for the two F_2_ populations (CLN4398-8 and CLN4397-4) derived from CLN4018F1-6-7U14-29-21-14-5 by ‘Hawaii 7996’ and CLN3641F1-5-11-14-4-25-20-11-7(F) by ‘Hawaii 7996’, respectively.

Population	Expected ratio	AUDPC ≤ 35 (resistant)	AUDPC > 35 (susceptible)	χ^2^-value	*P* value
CLN4018F1-6-7U14-29–21-14–5	1:0	30	0	–	–
Hawaii 7996	1:0	30	0	–	–
CLN4398-8	3:1	107	93	49.3	< 0.001
	9:7			0.6	0.4331
CLN3641F1-5-11-14-4-25-20-11-7(F)	1:0	30	0	–	–
Hawaii 7996’	1:0	30	0	–	–
CLN4397-4	3:1	117	43	0.3	0.5839
	9:7			18.5	< 0.001

### Validation of CAPS markers in two F2 populations confirmed resistant genes to bacterial wilt

To validate the identified polymorphisms, molecular markers were developed and first tested in the parental lines (CLN3641F1-5-11-14-4-25-20-11-7(F), CLN4018F1-6-7U14-29-21-14-5 and H7996) of our segregating populations (Table 4). Selection of polymorphisms for molecular marker development was based on the presence of the polymorphism in the highly resistant parent H7996 as well as location of polymorphisms within genes putatively associated with tolerance to stress (Supplemental Table 1). While the molecular markers developed here were polymorphic for the parental lines (data not shown), most markers were unable to accurately predict BW resistance phenotypes in the segregating F_2_ populations. Marker Bwr3.2dCAPS located on chromosome 3 was significantly associated with the phenotypic response in the CLN4398 population (Table 5). A minor QTL on chromosome 3 was previously found to contribute to resistance derived from H7996^8,9,28^. The reported size of *Bwr-3* is quite large, spanning most of the distal end of chromosome 3^9,28^and Bwr3.2dCAPS is within this region, supporting our results. Furthermore, marker Bwr3.2dCAPS was located within the *Asc g*ene (Solyc03g114600.4.1) which confers resistance to *Alternaria alternata* f. sp. *lycopersici* (AAL). The Bwr3.2dCAPS marker is based on the deletion of the 102^nd^ arginine in the *Asc* gene, resulting in a high-impact frameshift mutation that affects transcription and translation. The *Asc* locus was first identified by Gilchrist and Grogan^36^ and two alleles were found with resistance to the pathogen being dominant although the heterozygous condition conferred intermediate resistant phenotypes in AAL-toxin sensitivity assays. The *Asc* locus was later mapped to chromosome 3^37–39^ and was found to mediate resistance to sphinganine-analog mycotoxins (SAM)-induced apoptosis^40^. Interestingly, the homologous *LAG1-like Asc1* gene has been found to rescue tomato hair roots from SAM-induced cell death^41^ and the *Asc* gene has been found to be upregulated when plants were infested with *Bactericera cockerelli* infectious with *Candidatus Liberibacter solanacearum*^42^, potentially indicating *Asc* has multiple functions including response to bacterial infection and could be contributing to resistance to *Ralstonia* sp.

**Table 4:** Position (Mp), primer sequence, restriction enzyme required, and product size for the molecular markers developed and evaluated in this study for validation in the F_2_ populations.

Marker	Chromosome	Position (Mp)	Restriction enzyme	Primer (5′ → 3′)	Product size (R/S) (bp)
Bwr1.1indel	1	8.2	–	CAGGTAAGATGGAGAACATG	81/173
				TGTTCAATGTGCTGTTCGTG	
Bwr1.2HRM	1	8.5	–	GAGATTTCCTCAAGGTTTTCCTC	127
				AGCTTGTTTATCTCTCTCTC	
Bwr3.1HRM	3	0.6	–	CCACAGACAGATTTCTCGGT	126
				GTAGTGTCCAAGTAAGGTATAG	
Bwr3.2dCAPS	3	5.8	*BsrBI*	TTTGAATTTGTTGATCTTCTTCTCgCT	129/(105 + 24)
				ATTGATTTGGACGCGTGCTT	
Bwr4.1indel	4	2.0	–	GAGTGCGAGGAATGTATACT	(14 + 7 + 142)/(14 + 149)
				TCCAGTTTGTCTCATTTTCATCC	
Bwr4.2indel	4	2.0	–	CCAAGGTTTCGTGTATTTTAC	180/170
				TAATTGCAGCTTCCAAATGGAC	
Bwr4.3CAPS	4	2.0	*Ddel*	CTTGAGTTTCATATTTGCTAA	(18 + 46 + 105)/(64 + 105)
				GTGTCAACATTCTTATTGTA	
Bwr4.4HRM	4	2.7	–	TGAACCCTACATTCAGTAACTTTTTCCCAACA	150
				ATGGTTGTGGATGGCGGAG	
Bwr4.5HRM	4	59.0	–	TGCAGCAATACCTTTGGATAGGA	141
				CGCCACGCAATTTGAGACAG	
Bwr5.1HRM	5	2.2	–	TTCGCGTTTGAAGAAGAGGT	158
				TCGATTTTCGAACAAGCCTA	
Bwr7.1HRM	7	1.7	–	GAGATTTCCTCAAGGTTTTCCTA	159
				TCCCTTATCACTTAGGCCACA	
Bwr7.2HRM	7	1.89	–	TGCAACTTCCTTCCATTTTCCT	127
				TGCCCACAAATTCCATTCCA	
Bwr8.1CAPS	8	59.8	*NruI*	AGTCACACCAGATTGCAGGA	163/(132 + 31)
				GGGGATTTTCGAACGTTTAATGC	
Bwr9.1indel	9	0.3	–	CCAGCAAACCAAGTCGAT	220/161
				ATGGTCTTGTACTCAACTC	
Bwr9.2HRM	9	64.6	–	GATGTATGACAAGTCCAGTG	260
				GTGAGGCAAAGAACATACTTCCA

**Table 5:** Association between the phenotypic response when inoculated with the Pss4 isolate of bacterial wilt (race 1, biovar 3, *Ralstonia pseudosolanacearum*) of the CLN4398 F_2_ population and the Bwr3.2dCAPs molecular marker determined by Fisher’s Exact Test in R.

Population	AUDPC	R/H	S	*P* value
CLN4398-8	0–35	90	18	0.0178
	36–105	64	28	

## Conclusion

In this study, we utilized whole genome sequence data analysis, based on pairwise comparison of BW resistant and susceptible lines to identify candidate genes contributing to resistance above the levels conferred by *Bwr-6* and *Bwr-12*. Through this approach we found 27,046 SNPs and 5,975 indels specific to the resistant lines and causing high impact mutations in 385 genes. Furthermore, in addition to *Bwr-6* and *Bwr-12*, we found one or two independent loci contributed BW resistance based on inheritance patterns. Association between the phenotype and a newly developed molecular marker, Bwr3.2dCAPS in the previously reported *Asc* gene, was statistically significant but it did not completely explain the resistance phenotype. This study provides a basis for further investigations into new loci distributed throughout the genome that could contribute to BW resistance in tomato.

## Materials and methods

### Plant materials and inoculation

To identify highly resistant and susceptible individual plants for sequencing, six resistant tomato lines were selected (LS-89, H7997, F_7_-80-465-10-pink, F7-80-pink, H7996, and LE415 Anagha) and nine susceptible lines (CRA84-23-1 115, CRA84-57-1 140, T-245, S/T2, ‘Rodade’, LA3501, TBL-2, L390, and ‘Pant Bahar’), previously reported by Kunwar et al.^12^. The lines were inoculated with two virulent strains of *Ralstonia* sp., Pss4 (race 1, biovar 3^17^, *R*. *pseudosolanacearum*) and Pss1632 (race 3, biovar 2, *R*. *solanacearum*), representing the former designations of Phylotype I and Phylotype II, respectively. The bioassay was conducted during the hot season (June–July) of 2018 in a controlled environment greenhouse (19 ± 4 °C night and 39 ± 4 °C day) in Shanhua, Tainan, Taiwan (lat. 23.1°N; long. 120.3°E; elevation 12 m) and plants were fertilized weekly. The experiment followed a completely randomized design (CRD) with two replications, each with 20 plants for each of the strains used. The plants were inoculated at the 4–6 true leaf stage by drenching with a bacterial suspension (10^8^ CFU/ml) on the soil surface at a ratio of 1:10 (v:v) inoculum to potting mix. The individual plants were scored using a standardized scale twice a week for two weeks. The resistance percentage was calculated based on the number of asymptomatic plants during each time point. The highly resistant lines had a higher percent resistance after two weeks, while the highly susceptible lines had a low percent resistance within the first week after inoculation.

### DNA isolation, library preparation, and sequencing

For whole genome resequencing, five individual plants within each of the six resistant and nine susceptible lines were selected. Selection of plants was based on extremes in phenotype with susceptible individual plants selected based on early symptom occurrence, while resistant plants were selected by absence of symptoms at the final evaluation. DNA was extracted from each of the five plants using the Qiagen DNeasy kit following the manufacturer's instructions (Qiagen; Hilden, Germany), quantified using a fluorometer (Qubit 2.0, Invitrogen, Waltham, MA, USA) and pooled in equal amounts for each accession. The total DNA concentration, and DNA quality were determined using the TapeStation system (Agilent, Santa Clara, CA, USA). DNA libraries were generated using the NEBNext Ultra II DNA Library Prep Kit for Illumina (New England Biolabs, Ipswich MA, USA) according to the manufacturer's instructions. The quality of the libraries was assessed using the TapeStation system with D1000 High Sensitivity ScreenTape. Next-generation sequencing using the HiSeq Illumina platform with 150 bp paired-end reads was conducted by Welgene Biotech Co., Ltd. (Taipei, Taiwan). Total DNA was isolated from leaf tissue collected prior to inoculation and stored at −80 °C until the phenotyping experiment was completed.

### Sequence analysis

For the whole genome sequencing analysis, the quality of reads was checked using FastQC (v. 0.11.7)^43^. All reads were trimmed based on an average Phred quality score of 20 for 4 consecutive bases and we discarded reads shorter than 50 bp using Trimmomatic (v.0.36)^44^. We then mapped the reads to the annotated ‘Heinz 1706’ reference genome (v.SL4.0)^45^ using the “mem” algorithm of Burrows-Wheeler Alignment (BWA-MEM; v0.7.17)^46^ and the average number of reads was 1.15 × 10^8^. Minimum coverage depth was set to 25 × , but most of the time mean read depth was ~ 50 × .

### Variant calling

Variant calling was performed using Genome Analysis Toolkit (GATK; v4.1.6.0)^47^ the Picard Toolkit (v2.21.9)^48^ and samtools (v1.10)^49^. First, PCR duplicates were removed using MarkDuplicates for each sample and then HaplotypeCaller, GenotypeGVCFs, and VariantFiltration sequentially were used for variant calling, the filtration of variants to get the first version of homozygous SNP, and indels. For the filters in VariantFiltration, there were six filters for SNPs and three for indels. For SNPs, SNPs with FisherStrand (FS) equal to or less than 60, StrandOddsRatio (SOR) equal to or less than 3, RMSMappingQuality (MQ) equal to or greater than 40, MappingQualityRankSumTest (MQRankSum) equal to or greater than -12.5 and ReadPosRankSum (ReadPosRankSum) equal to or greater than -8.0 were retained. For indels, variants with FS equal to or less than 200, ReadPosRankSum equal to or greater than -20. We used the threshold QualByDepth (QD) as equal to or greater than 2 for both SNPs and indels were kept. The first version of homozygous variants was used to recalibrate the bam files of each sample using BaseRecalibrator and BQSR, then variant calling was again performed based on recalibrated bam files to get the final version of homozygous SNPs and indels written in the Variant Call Format (VCF) files. SNPs with read depth > 10, no missing data, and no heterozygous sites were retained, resulting in about 1.8 million SNPs. These SNPs were then used to calculate the. Principal Coordinate Analysis (PCA) of the genetic distance with TASSEL 5.0 and in R-3.6.3^50^.

A customized script in R-3.6.3 was developed to compare the variants of six resistant lines with nine susceptible lines. To comprehensively screen the candidate markers that contributed to the resistance, each resistant line was compared individually with all susceptible lines and only variants polymorphic between the individual resistant lines and all susceptible lines were retained. Then, the variant annotation and effect prediction based on these variants only from six resistant lines was performed using SnpEff 4.3t^51^. The distribution of variants and highly affected genes were visualized by Circos (v 0.69–8)^52^.

### Molecular marker development

Based on the polymorphisms specific to resistant lines with high impact differences in predicted effects, nine loci predicted to encode proteins with putative functions associated with resistance to bacterial wilt were selected. In each selected locus, molecular markers were designed to test for associations between the sequence polymorphism in candidate genes and the resistant phenotype, which could not be explained by *Bwr-6* and *Bwr-12* QTLs. A total of 15 molecular markers were designed for validation, eight high resolution melting (HRM) markers, four insertion-deletion (indel) markers, two cleaved amplified polymorphic sequence (CAPS) markers, and one derived cleaved amplified polymorphic sequence (dCAPS) marker. All molecular markers were first used to genotype the parental lines and only those that were confirmed to be polymorphic were selected to genotype the validation populations. For the gel-based molecular markers, the PCR reactions included 2 μL DNA, 2 μL 10 × PCR buffer with 1.5 mM MgCl2 (10 × GOLD Buffer), 0.15 mM dNTPs, 0.25 U Taq polymerase (Gold Taq 250 U) and 0.5 mM for forward and reverse primers. The PCR temperature profile was as follows: 95 °C for 10 min, 35 cycles for 95 °C for 30 s., 55 °C for 45 s. and 72 °C for 45 s., followed by 72 °C for 5 min and final hold at 15 °C. The PCR product were separated on 6% polyacrylamide gels alongside a 50-bp DNA ladder in TBE buffer (90 mM Tris, 90 mM Boric acid, 2 mM EDTA, pH 8.4, VWR) at 160 V and 400 mA for 30–55 min. The polyacrylamide gels were stained by DNA fluorescent dye (FluoroStainTM DNA Fluorescent Staining Dye; Green, 5,000X, SMOBIO) for 10 min. The stained polyacrylamide gels were visualized using a blue-light imaging system (BIO-1000F). For the HRM molecular markers, the reactions were performed using a total volume of 20 μL containing 20 ng of PCR fragment on a Corbett Rotor Gene 6000. The reaction used the SensiFAST™ HRM Kit and followed the manufacturer's instructions. For PCR, 5 min pre- denaturation at 95 °C was followed by 50 cycles of 95 °C for 10 s, 60 °C for 30 s, and 72 °C for 35 s. For the HRM analysis, the amplicons spanned from 65 to 95 °C, rising by 0.1 °C each step. The Rotor-Gene Q software version v2.2 was used to analyze the melting curve results.

### Validation

For marker validation, two F_2_ populations coded CLN4397-4 (CLN3641F1-5-11-14-4-25-20-11–7(F) × H7996 [160 individuals]) and CLN4398-8 (CLN4018F1-6-7U14-29-21-14-5 × H7996 [200 individuals]) were developed, all of which were homozygous for both the *Bwr-6* and *Bwr-12* QTLs except for a few heterozygotes in the CLN4398 population. All lines, including one susceptible check (L390) and parental lines, were grown in the greenhouse as previously mentioned and fertilized weekly. At the 4–6 true leaf stage, the F_2_ populations were screened with the Pss4 strain by drench inoculation as described above. Plants were scored using a standardized 0 to 5 rating scale twice weekly for two weeks after inoculation. The scores were used to calculate the area under the disease progress curve (AUDPC) and the deviation from expected segregation ratios of resistance in the two F_2_ populations was determined using the χ^2^ test in R-3.6.3^50^.

Sequencing data were submitted to the National Center for Biotechnology Information (NCBI) Sequence Read Archive (SRA).

### Ethical statement

Experimental research and field studies on plants (either cultivated or wild), including the collection of plant material, complies with relevant institutional, national, and international guidelines and legislation.

## Supplementary Information

Supplementary Information 1.

Supplementary Information 2.

Supplementary Information 3.

Supplementary Information 4.

Supplementary Information 5.

Supplementary Information 6.

## Data Availability

The Illumina sequencing data have been deposited at NCBI under BioProject PRJNA725647. (reviewer linkhttps://dataview.ncbi.nlm.nih.gov/object/PRJNA725647?reviewer=d15n1ajijjhsspov22ta9s50fa) All other data are available at the World Vegetable Center repository, HARVEST (worldveg.org/harvest3).
