# Supplementary figures and images for: Whole genome resequencing and complementation tests reveal candidate loci contributing to bacterial wilt (Ralstonia sp.) resistance in tomato
Source: Sci Rep. 2022 May 19;12:8374. doi: 10.1038/s41598-022-12326-x (PMC9120091; doi:10.1038/s41598-022-12326-x)

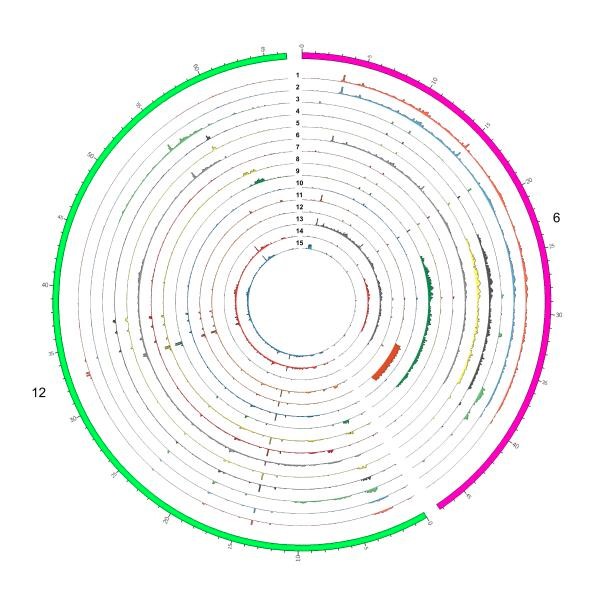

Supplement: Supplementary file 1 — Supplementary Information 1. [file 41598_2022_12326_MOESM1_ESM.jpg]

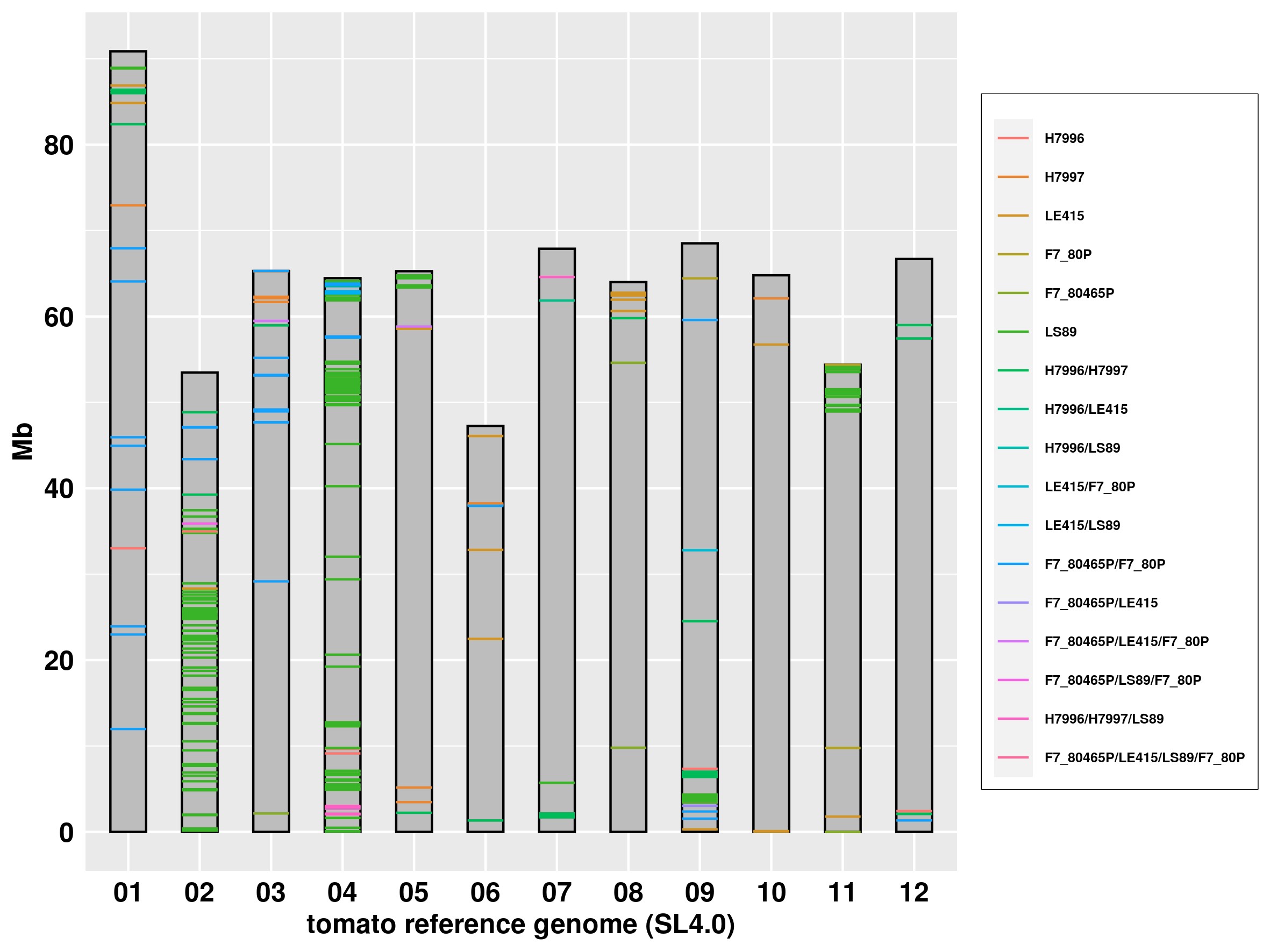

Supplement: Supplementary file 2 — Supplementary Information 2. [file 41598_2022_12326_MOESM2_ESM.jpg]
