# Supplementary material for: Whole genome resequencing and complementation tests reveal candidate loci contributing to bacterial wilt (Ralstonia sp.) resistance in tomato
Source: Sci Rep. 2022 May 19;12:8374. doi: 10.1038/s41598-022-12326-x (PMC9120091; doi:10.1038/s41598-022-12326-x)
Supplement: Supplementary file 3 — Supplementary Information 3. [file 41598_2022_12326_MOESM3_ESM.docx]

|  | LS-89 | Hawaii 7996 | Hawaii 7997 | LE415 | F7_80P | F7_80465P |
| --- | --- | --- | --- | --- | --- | --- |
| Type | | | | | | |
| SNPs | 313359 | 26426 | 27770 | 19222 | 30944 | 30870 |
| InDel | 42444 | 6185 | 6424 | 4471 | 6605 | 6192 |
| Regions | | | | | | |
| Intergenic regions | 315391 | 26696 | 27740 | 19433 | 33614 | 33105 |
| 5’UTR | 1363 | 212 | 246 | 158 | 120 | 120 |
| 3’UTR | 2291 | 367 | 404 | 356 | 318 | 313 |
| Effects on amino acid | | | | | | |
| Synonymous mutation | 2910 | 485 | 537 | 340 | 231 | 236 |
| Non-synonymous mutation | 3615 | 522 | 568 | 377 | 407 | 405 |
| High impact polymorphisms | | | | | | |
| Lost or gained top sites | 101 | 7 | 20 | 11 | 17 | 14 |
| Lost start sites | 22 | 3 | 3 | 4 | 2 | 2 |
| Frame shift | 204 | 46 | 43 | 24 | 35 | 33 |
| The donor or acceptor of splice sites | 52 | 8 | 8 | 4 | 11 | 11 |
