# Supplementary material for: Whole genome resequencing and complementation tests reveal candidate loci contributing to bacterial wilt (Ralstonia sp.) resistance in tomato
Source: Sci Rep. 2022 May 19;12:8374. doi: 10.1038/s41598-022-12326-x (PMC9120091; doi:10.1038/s41598-022-12326-x)
Supplement: Supplementary file 6 — Supplementary Information 6. [file 41598_2022_12326_MOESM6_ESM.docx]

Supplemental Figure 1. The distribution of SNPs on chromosomes 6 and 12 among the 15 bacterial wilt (*Ralstonia* sp.) resistant and susceptible tomato lines. The histograms represent the number of SNPs in 100-kb for the 15 tomato accessions. The lines are numbered 1) Hawaii 7996, 2) Hawaii 7997, 3) LE415, 4) F7_80P, 5) F7_80465P, 6) LS89, 7) Bahar, 8) CRA84_115, 9) CRA84_140, 10) L390, 11) LA3501, 12) Rodade, 13) ST2, 14) T_245, and 15) TBL_2.

Supplemental Figure 2. The genome-wide distribution of genes highly affected by polymorphisms of six bacterial wilt (*Ralstonia* sp.) resistant tomato lines. The horizontal bars on each chromosome represent their coordinates, and the colors on the legend show the corresponding resistant accessions of bars on each chromosome.

Supplemental Table 1. Characteristics of the polymorphisms for six bacterial wilt (*Ralstonia* sp.) resistant tomato lines.

Supplemental Table 2. Location, sequence, and predicted effect information for the high impact polymorphisms identified for the six bacterial wilt (Ralstonia sp.) resistant tomato lines.

Supplemental Table 3. Presence of previously developed molecular markers associated with *Bwr-6* (SLM6-17, SLM6-94, SLM6-110, SLM6-118, SLM6-124) and *Bwr-12* (SLM12-2, SLM12-10) in the bacterial wilt (*Ralstonia* sp.) resistant tomato parental lines (Hawaii 7996, CLN3641F1-8-11-14-4-25-20-11-7(F), and CLN4018F1-6-7U14-29-21-14-5) and F_2_ progeny of the two populations (CLN4397-4 and CLN4398-8).
